# Perioperative microcirculatory monitoring using handheld video microscopy: a proof-of-concept observation

**DOI:** 10.1016/j.bjane.2025.844660

**Published:** 2025-07-05

**Authors:** Rafael M. Linhares, Eduardo Tibiriça

**Affiliations:** a Universidade Federal do Estado do Rio de Janeiro, Rio de Janeiro, RJ, Brazil; b Ministério da Saúde, Instituto Nacional de Cardiologia, Departamento de Pesquisa e Ensino, RJ, Brazil

Microcirculation exhibits a remarkable capacity for adaptation to its cellular surroundings and can autoregulate, thus preserving a constant blood flow that remains unaffected by alterations in systemic blood pressure under physiological conditions.^1^ In this context, hemodynamic coherence plays a crucial role in maintaining homeostasis and supporting proper organ function.^2^ Nevertheless, various factors may lead to disconnection between macro- and microcirculation and subsequent tissue damage.^3^ Notably, microcirculatory alterations can occur even when global systemic hemodynamics are preserved, resulting in the functional decoupling of macrocirculation and microcirculation, a phenomenon also known as “hemodynamic incoherence”.^2^ These factors include sepsis or shock, alterations in blood viscosity and shear stress, and iatrogenic injury.^4^

The perioperative setting is complex, involving diverse patient populations, varying illness severity, and differences in surgical and anesthetic approaches. In the intraoperative phase, various factors, such as surgical procedures, bleeding, low body temperature, and the administration of anesthetics and vasopressors, can lead to changes in the microcirculation and reduced blood flow to tissues. However, improvements in systemic hemodynamic parameters may not always lead to a corresponding improvement in microcirculatory flow.^2^ The apparent effects of anesthesia on the vascular system may cause changes in microcirculation and, consequently, in tissue parameters and oxygenation. The induction of anesthesia, for example, diminishes capillary red blood cell flow, as evidenced by reduced red blood cell velocity and a smaller proportion of perfused vessels, while simultaneously increasing capillary vessel density.^5^ In this context, the key objective of perioperative microcirculation monitoring is to guide therapeutic interventions that specifically target the microcirculation. Nevertheless, there is a lack of specific studies on point-of-care (bedside) testing with minimally invasive devices, such as handheld cameras, in patients under general anesthesia.

The objective of this preliminary study was to establish the feasibility of the sublingual microcirculation monitoring method in a clinical surgical scenario under general anesthesia. Accordingly, we report the case of a patient who underwent laparoscopic cholecystectomy surgery. In this study, we evaluated the microcirculation status using a real-time, noninvasive, point-of-care microcirculatory imaging technique. More precisely, we utilized an incident dark field camera (Fig. 1) to examine sublingual microcirculation. The Cytocam-IDF is a third-generation handheld microscope that features a high-density pixel-based imaging chip and a short-pulsed illumination source controlled by a computerized system. This system allows serial measurements to be made without the need to refocus, an important feature compared with previous generation devices that require time-consuming manual adjustment of focus controls. The IDF-Cytocam imaging system allows a direct and noninvasive view of microvessels up to a diameter of 50 µm, including arterioles, capillaries, and venules.^6^ Importantly, the validity of this imaging technique has been previously established and documented.^7^^,^^8^ Moreover, previous research has shown that the sublingual region has a uniform spatial distribution, allowing the assessment of several microvascular parameters, such as total and functional vascular density.^9^ The video microscopic assessment of mucosal sublingual microcirculation has been considered a sensitive indicator of systemic microvascular alterations, including circulatory failure.^10^

**Figure 1:**
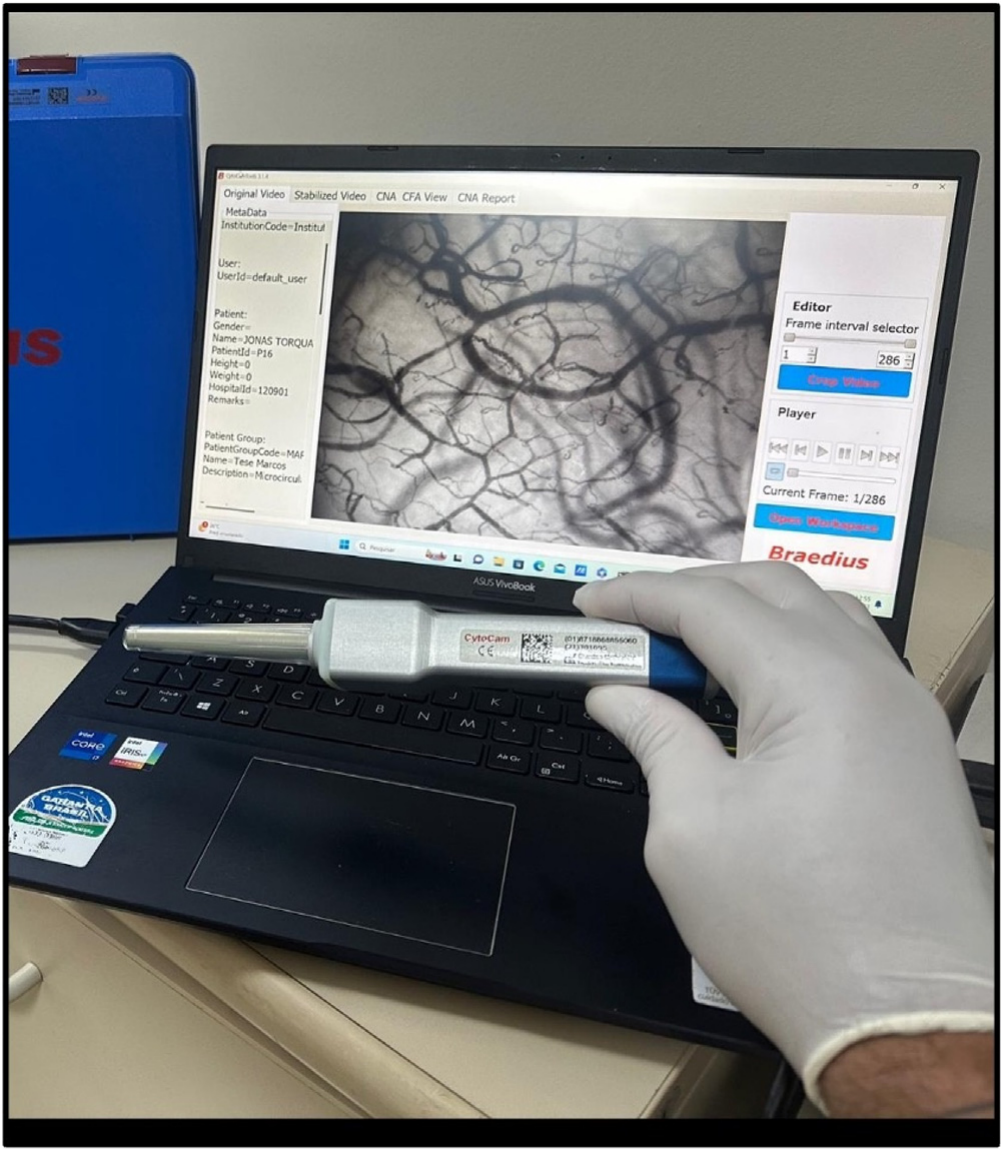
The CytoCam-IDF handheld video microscope used for the visualization of microcirculatory parameters in our work, which is based on Incident Dark Field (IDF) technology. The CytoCam is a pen-like device and is held as such. The low weight of the device (120 g) minimizes pressure artifact problems that were present in earlier heavy devices. The camera is connected to a device controller based on a medical-grade computer or a suitable portable device, such as a laptop or tablet, which is used for image storage (from https://braedius-medical.com/products/).

Given that the handheld device is still in experimental use and could be established as a perioperative monitoring device, the recommendation is to test it only in patients with steady cardiovascular systems. This specific case seeks to demonstrate the viability of the approach for monitoring perioperative microcirculation without assuming a positive postoperative result. The subject read and signed a specific informed consent form concerning the present publication.

It is important to note that our study is a proof-of-concept investigation. As a small-scale, preliminary study, its primary goal is to assess the feasibility and viability of a new methodology for evaluating tissue perfusion by examining systemic microcirculation using handheld camera-based technology. Additionally, this study could help identify potential challenges or limitations associated with this methodology.

A 74-year-old female (BMI 32.0 kg.m^-2^) with hypertension and hypothyroidism (ASAII) was diagnosed with cholelithiasis and underwent uneventful laparoscopic cholecystectomy. The patient did not present any pre-existing vascular condition that could alter microcirculatory responses. Moreover, the patient did not present any sign of dehydration and received IV infusion of 5 mL.kg^-1^.h^-1^ ringer lactate during surgery (total 1,200 mL). The anesthetic technique utilized was balanced veno-inhalation. Induction was performed with 240 µg fentanyl (3 µg.kg^-1^), 160 mg propofol (2 mg.kg^-1^), 50 mg rocuronium (0.6 mg.kg^-1^) and 120 mg lidocaine (1.5 mg.kg^-1^). After orotracheal intubation with a videolaryngoscope (King Vision, King Systems, Noblesville, USA), anesthesia was maintained by sevoflurane (0.5 MAC), an inhalational anesthetic agent, plus dexmedetomidine infusion (0.5 µg.kg^-1^.h^-1^) initiated at the beginning of induction.

Anesthetic depth was considered as maintained when the Bispectral Index™ (BIS™) monitoring system values (Medtronic, Watford, United Kingdom) remained between 40 and 60 and when the mean arterial pressure and heart rate remained within 10%–20% of their preoperative values; the use of vasopressors was not necessary during surgery. The total anesthesia and surgical times were 180 and 135 minutes, respectively. Neuromuscular blockade was reversed at the end of the surgical procedure through intravenous administration of sugammadex (2 mg.kg^-1^).

A microcirculatory evaluation was performed four times: before anesthesia induction (time 1), 45 min (time 2), and 60 min (time 3) after starting dexmedetomidine infusion, and after anesthetic recovery (time 4). The peak effect of the continuous infusion of dexmedetomidine is known to occur between 45 and 60 minutes. At each time evaluation, we took five videos (5-sec duration) for further analysis. In the laboratory, we selected the top three video quality scores and extracted some microcirculation data using Cytocam Tools 3.1.4 software (Braedius Medical, Huizen, The Netherlands). We consider that the most clinically relevant microvascular parameters are total number of capillary vessels and capillary vessel density, because they reflect tissue perfusion and oxygenation. The key microcirculatory parameters obtained are summarized in Table 1.

**Table 1:** Analysis of key microvascular parameters in the sublingual region, assessed using a handheld Incident Dark-Field (IDF) imaging camera at baseline, 45 and 60 minutes after the initiation of dexmedetomidine infusion, and following anesthetic recovery. The evaluation included capillary vessels (diameter: 6–16 µm), non-capillary vessels (diameter: 16–50 µm), and the total vessel count, encompassing all microvessels with a diameter of less than 50 μm.

	Baseline	45 min	60 min	Recovery
**Total number of capillary vessels (n)**	686	888	1058	563
**Capillary vessel density (mm.mm^-2^)**	11.42	13.74	17.28	10.77
**Total number of noncapillary vessels (n)**	89	97	112	73
**Noncapillary vessel density (mm.mm^-2^)**	1.09	1.08	1.47	1.16
**Total vessel number (n)**	764	972	1170	636

We found a marked increase in the total capillary number, capillary vessel density and total number of microvessels at times 2 and 3, and a slighter increase in the number and density of noncapillary vessels. Some prospective randomized studies with the same microcirculation evaluation methods reported similar results under dexmedetomidine infusion.^11^^,^^12^ In these studies, researchers included patients undergoing on-pump coronary artery bypass graft and compared the single use of dexmedetomide, or in association with propofol infusion, and found improved sublingual microcirculation indices. An interesting point is that cardiopulmonary bypass is known to impair sublingual microcirculation.^11^

The primary focus of hemodynamic monitoring in conventional practice is directed toward the assessment of macrocirculatory parameters. This paper indicates the potential for a new procedure better suited for intraoperative situations. However, it is important to note that the macrocirculatory profile may not accurately represent tissue perfusion. Even with adequate blood pressure and cardiac output, peripheral tissues may experience insufficient perfusion, leading to ischemia and organ dysfunction.^2^

Conversely, confirming the condition of the microcirculatory environment may help prevent unwarranted increases in the use of vasopressor medications and other measures that could cause injury in individuals with low blood pressure. A better understanding of perioperative microcirculatory dynamics could improve individualized hemodynamic management and optimize patient outcomes.

Although handheld vital microscopes remain experimental for systemic microcirculatory assessment, they hold potential for future integration into routine perioperative monitoring by physicians and nurse specialists. Cytocam, the handheld microcamera used in the present study, enables the visualization of microcirculation at the tissue level, which is crucial during anesthesia. By providing clear, real-time imaging of capillaries, arterioles, and venules, it could help anesthesiologists closely monitor the effects of anesthesia on circulation and oxygenation with great precision. Additionally, it aids in assessing tissue perfusion and detecting early signs of ischemia or hypoxia, both of which are critical to monitor during anesthesia, particularly in high-risk surgeries or for patients with complex medical conditions.

In terms of research applications, Cytocam can be employed in clinical studies to examine how anesthesia impacts vascular function, especially in relation to specific anesthetic agents or techniques. It can also be used to explore how various confounding factors (e.g., age, comorbidities) influence the response to anesthesia, paving the way for the development of more personalized, patient-specific anesthetic protocols. Furthermore, in drug testing and evaluation for pharmaceutical studies, Cytocam offers a valuable tool for assessing the effects of new anesthetic or vasodilatory drugs on microvascular function, thus supporting the creation of safer and more effective anesthetic agents.

## Limitations of the study

This study is a proof-of-concept report, with the primary goal of presenting a novel and useful device for use in the perioperative period. As this is a single case, it does not include a control group. These data will be presented in a clinical trial when available. This case involves a routine cholecystectomy, a procedure that typically has favorable postoperative outcomes. The key value of this report lies in demonstrating the effectiveness of the method in evaluating systemic microcirculation in a patient with a stable cardiovascular status.

While Cytocam offers significant advantages in monitoring microcirculation during anesthesia, its use is also associated with several limitations and challenges, including technical issues, reproducibility concerns, and constraints in broader clinical applicability. Image quality can be affected by factors such as camera positioning and patient movement, potentially leading to blurry or distorted visuals, especially if the device is not held correctly or if the patient moves during the procedure.

Effective use of Cytocam requires technical proficiency, as anesthesiologists and medical staff must be trained to operate the device properly and accurately interpret the images. Additionally, the cost of acquiring and maintaining Cytocam can be expensive, particularly for smaller hospitals or healthcare facilities with limited budgets, potentially restricting its use to well-funded academic or research institutions.

In conclusion, further clinical validation and improvements in availability will be crucial for the widespread adoption of Cytocam in anesthesia and other medical fields. In this regard, a clinical study is currently underway within our research team to evaluate the systemic microvascular effects of continuous dexmedetomidine infusion, using Cytocam in the sublingual region, in low-risk patients undergoing laparoscopic cholecystectomy. The present proof-of-concept report demonstrates that monitoring systemic microcirculation using a handheld, microcamera-based technology in the sublingual region is feasible during surgery under general anesthesia. The observed increases in the number and density of microvessels during anesthesia maintenance, followed by a return to baseline values during recovery, suggest that this technology is reliable for evaluating microvascular perfusion during anesthetic procedures.

## Availability of data and material

The datasets used and/or analyzed during the current study are available from the corresponding author upon reasonable request.

## Authors' contributions

RML and ET contributed to the conception and design of the study and to the analysis and interpretation of the data; and RML and ET were involved in the drafting of the manuscript and the literature review. Both authors have approved the final version to be published and are publicly responsible for its content.

## Conflicts of interest

The authors declare no conflicts of interest.

## Appendix Supplementary materials

Image, application 1

Image, video 2

Image, video 3
