# Supplementary material for: Perioperative microcirculatory monitoring using handheld video microscopy: a proof-of-concept observation
Source: Braz J Anesthesiol. 2025 Jul 5;75(6):844660. doi: 10.1016/j.bjane.2025.844660 (PMC12336682; doi:10.1016/j.bjane.2025.844660)
Supplement: Supplementary file 1 [file mmc1.docx]

**BJAN-D-25-00064_Supplementary Material**

**Video 1** An illustrative recording of the patient's sublingual microcirculation, assessed using Cytocam-IDF imaging.

**Video 2** An illustrative recording of the patient's sublingual microcirculation, assessed using Cytocam-IDF imaging, with capillary vessels highlighted by blue dotted lines.
